# Evaluating the use of non‐invasive hair sampling and ddRAD to characterize populations of endangered species: Application to a peripheral population of the European mink

**DOI:** 10.1002/ece3.10530

**Published:** 2023-09-18

**Authors:** Alfonso Balmori‐de la Puente, Lídia Escoda, Ángel Fernández‐González, Daniel Menéndez‐Pérez, Jorge González‐Esteban, Jose Castresana

**Affiliations:** ^1^ Institute of Evolutionary Biology (CSIC‐Universitat Pompeu Fabra) Barcelona Spain; ^2^ Biosfera Consultoría Medioambiental S.L. Oviedo Spain; ^3^ DESMA Estudios Ambientales Ituren (Navarra) Spain

**Keywords:** ddRAD, inbreeding, individualization, *Mustela lutreola*, non‐invasive samples, relatedness, spatial capture‐recapture

## Abstract

The application of next‐generation sequencing (NGS) to non‐invasive samples is one of the most promising methods in conservation genomics, but these types of samples present significant challenges for NGS. The European mink (*Mustela lutreola*) is critically endangered throughout its range. However, important aspects such as census size and inbreeding remain still unknown in many populations, so it is crucial to develop new methods to monitor this species. In this work, we placed hair tubes along riverbanks in a border area of the Iberian population, which allowed the genetic identification of 76 European mink hair samples. We then applied a reduced representation genomic sequencing (ddRAD) technique to a subset of these samples to test whether we could extract sufficient genomic information from them. We show that several problems with the DNA, including contamination, fragmentation, oxidation, and possibly sample mixing, affected the samples. Using various bioinformatic techniques to reduce these problems, we were able to unambiguously genotype 19 hair samples belonging to six individuals. This small number of individuals showed that the demographic status of the species in this peripheral population is worse than expected. The data obtained also allowed us to perform preliminary analyses of relatedness and inbreeding. Although further improvements in sampling and analysis are needed, the application of the ddRAD technique to non‐invasively obtained hairs represents a significant advance in the genomic study of endangered species.

## Graphical abstract

In this work, hair tubes were placed along river banks in a border area of the Iberian population of European mink. The hairs obtained from the target species using these traps were used to construct a ddRAD library. Various bioinformatic techniques were used to reduce the problems associated with this type of non‐invasive sampling, allowing us to reliably genotype individuals in the area and perform preliminary analyses of relatedness and inbreeding.

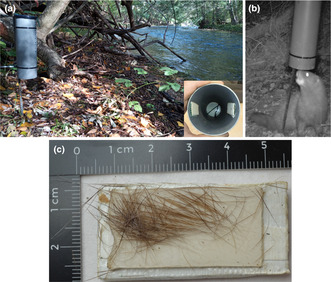

## 1 INTRODUCTION

The genetic analysis of non‐invasive and minimally invasive samples is important for the study of wildlife populations because it allows their monitoring and the assessment of their genetic parameters without the need to capture or manipulate the animals (Carroll et al., 2018; Schwartz et al., 2007). In particular, hair sampling devices have been shown to be effective for genetic analysis of mammalian populations (Roche, 2008). However, non‐invasive samples present significant challenges for genetic and genomic studies, as these samples are often degraded and can be contaminated with bacteria and other sources of exogenous DNA (Carroll et al., 2018; Pompanon et al., 2005; Taberlet et al., 1999).

Because of the difficulties with non‐invasive samples, target enrichment, SNP arrays, and other next‐generation sequencing (NGS) techniques have been used to capture endogenous DNA using different types of probes and primers. In conservation studies, these techniques typically generate one or two hundred single nucleotide polymorphisms (SNPs), which have been shown to be sufficient to individualize samples (Carroll et al., 2018; Srivathsa et al., 2021). However, more SNPs are generally needed for further genetic analysis of individuals and populations. A cost‐effective NGS technique that can produce several hundred to thousands of SNPs is double digest restriction site‐associated DNA (ddRAD; Peterson et al., 2012), which has been shown to be effective in performing relatedness, connectivity, and inbreeding analyses, of great interest in conservation genomics studies (Escoda et al., 2019; Prost et al., 2022). This technique has also been successfully applied to feces following methylation‐based enrichment of vertebrate DNA (Tyagi et al., 2022). However, ddRAD has not yet been widely applied to other types of non‐invasive samples, such as hair obtained from hair traps. Unlike feces, which typically contain a large amount of exogenous DNA (Perry et al., 2010), hair samples are expected to contain a higher proportion of endogenous DNA. However, degradation may prevent their use for genomic studies. Therefore, it is not well known how the small amounts of raw material available in hair, as well as DNA contamination, fragmentation, and chemical degradation, affect the quality of the obtained SNPs and the downstream analyses. These aspects are well understood in other fields, such as ancient DNA, where important goals have been achieved with some very old samples (Briggs et al., 2007). It would then be essential to advance the development of a genomic technique such as ddRAD for its use with non‐invasively obtained hair samples, but it is necessary to first verify that the problems associated with these samples do not compromise the quality and interpretation of the data.

The European mink (*Mustela lutreola*) is a semi‐aquatic mammal whose populations have declined sharply throughout its European range in recent years, making it one of the most endangered mammal species in the world (Maran et al., 2016; Skorupski, 2020). The main threats to the European mink include competition with the invasive American mink (*Neovison vison*), extremely low genetic diversity, and degradation of its riparian habitat (Cabria et al., 2015; Palazón & Melero, 2014). Therefore, it is crucial to monitor European mink populations in different areas to determine their population size, degree of connectivity, and other important parameters for management and conservation. However, it is a very elusive species and difficult to detect. To date, the main method used to census this species has been live trapping, which is costly and causes significant disturbance to captured individuals. More recently, camera trapping, hair tubes with morphological hair identification, and environmental DNA from water samples have been employed to detect the presence of this species (Croose et al., 2023). However, techniques that allow the individualization of non‐invasive samples are not yet available for this species. Applying genomic techniques to non‐invasive hair samples would enable more detailed and informative analyses, which would be highly valuable for the management and conservation of this endangered species.

In this study, we aimed to apply a ddRAD method to non‐invasive hair samples from a European mink population in the Iberian Peninsula, at the southwestern limit of its distribution range. The main goal was to test whether sufficient genomic information could be obtained from these samples for individualization, relatedness, and inbreeding analyses. An important methodological effort was made to optimize specific field, laboratory, and bioinformatics protocols in order to detect and address potential problems due to sample degradation. We found that, after applying these methods, it was possible to genotype samples and perform relatedness and inbreeding analyses in the population. Finally, we propose several procedures that should be applied in genomic analysis of non‐invasive samples to avoid artifacts and problems in the interpretation of the results.

## 2 MATERIALS AND METHODS

### 2.1 Hair traps, study area, and sampling effort

**FIGURE 1:**
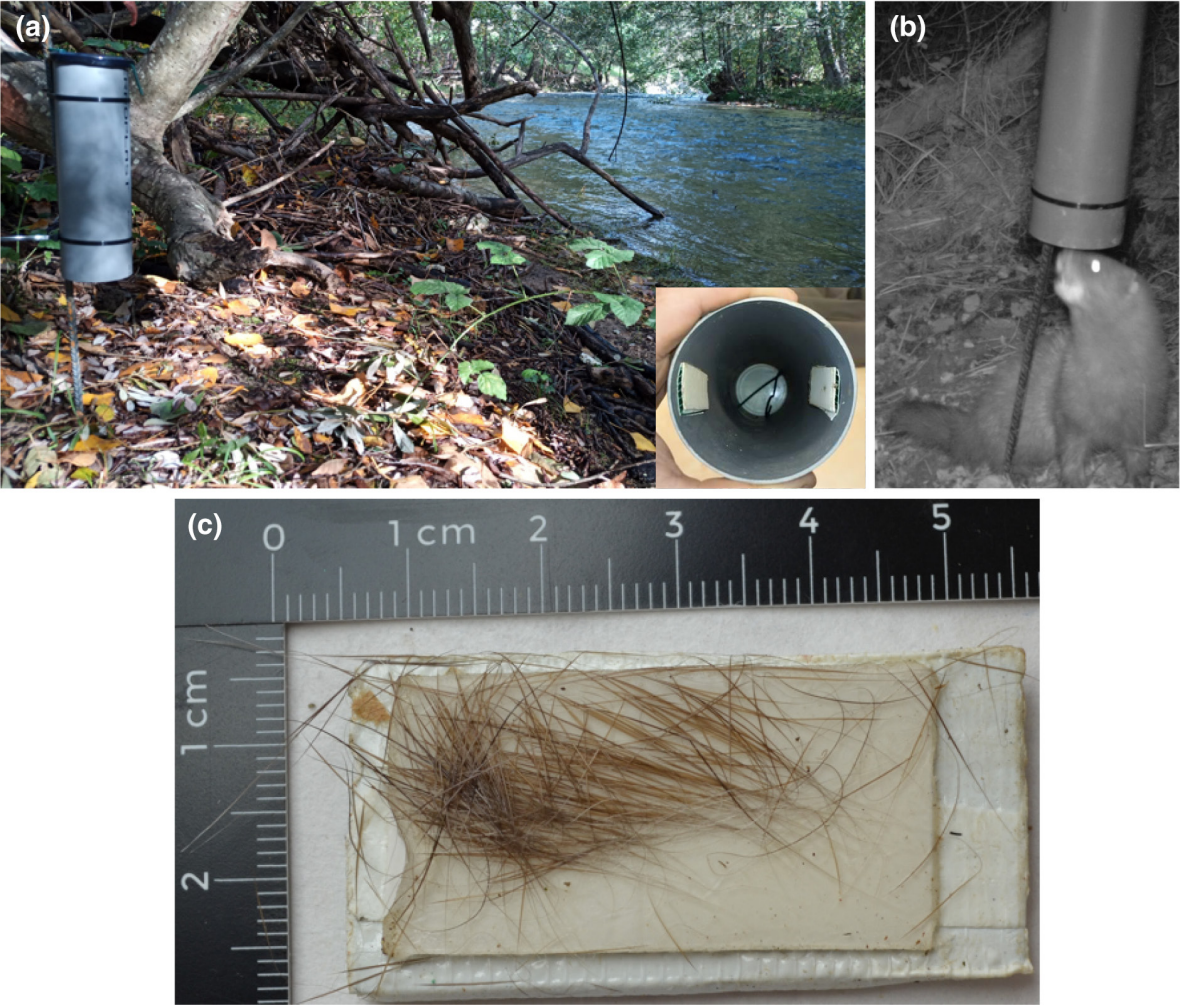
(a) Image of a hair trap placed in the field with the lower right inset showing the arrangement of the adhesive sheets inside the trap. (b) European mink approaching the hair trap. (c) Adhesive sheet with European mink hairs attached to it.

Previously developed hair tube traps (Roche, 2008) were optimized to collect hair samples from the European mink. We used single‐tunneled vertical PVC tubes (7.5 × 25 cm) with an attractant next to the upper end and two adhesive sheets (2 × 4 cm), commonly used in small mammal traps (STV182 International Ltd.; Solway Feeders Ltd.), attached in the lower portion. As an attractant, we used chicken bait, proved to be the most effective in preparatory studies. The traps were placed close to the vegetation in the vicinity of watercourses, with the bottom left open and 15 cm off the ground, which facilitated access by the European mink and prevented larger mammals from easily reaching the traps (Figure 1a,b).

**FIGURE 2:**
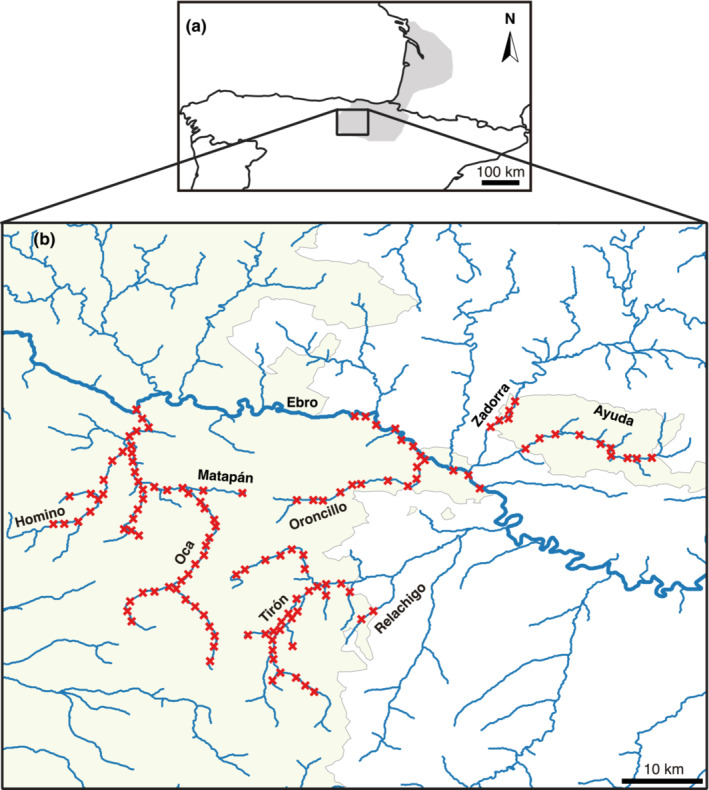
(a) Map of the northern Iberian Peninsula and southern France showing the western range of the European mink. (b) Study area showing the 127 trap localities. The province of Burgos, where the study was carried out, is shown in light green. The names of the rivers where the traps were placed are indicated, and the Ebro River, the largest river in the area, is marked with a thicker line.

The study area is located in the north‐central Iberian Peninsula (province of Burgos, Castilla y León) and covers 311 km of waterways in the Ebro River basin (Figure 2a,b). This population is located at the southwestern limit of the species distribution range. The area was colonized in the last few decades but has maintained a low population density over the years (Palazón et al., 2003; Palazón & Gómez, 2007). Sampling was carried out over a 3‐week period in March 2020. Hair traps were placed along river sectors with suitable habitat for the species and where the species had been detected in previous years (Figure 2b). A total of 254 hair traps were placed at 127 locations separated by approximately 2 km, with two traps per locality placed about 100 m from each other (in this work, we refer to a locality as the place with the two traps). The traps were checked on three occasions, at 7‐day intervals. The adhesive sheets containing hairs (Figure 1c) were carefully removed and taken to the laboratory, where they were kept frozen.

Once in the laboratory, the hairs attached to each of the adhesive sheets were removed with forceps, and only samples morphologically attributed to mustelids (Debrot et al., 1982) were used for the subsequent genetic analyses (Table S1 in Appendix S1). Thus, each sample included all hairs from one adhesive sheet. The number of hairs per sample ranged from 1 to more than 100. The hairs were transferred to ethanol tubes for storage at −20°C. For genotyping, one positive sample per locality and trap check was normally used. However, if the DNA concentration was low (see below), more samples per locality and trap check were used to increase the chance of success.

In addition, tissue samples (a small piece of ear cartilage) from 15 European mink were processed and used to detect endogenous sequences from the non‐invasive samples and to identify individuals for which both tissue from live‐trapping and hair from hair traps were available. The tissue samples had been collected in works coordinated by the regional government of Castilla y León through live trapping in the same area in recent years (2017–2020; Table S2) and were loaned for this study.

No animals were specifically handled or captured for this work and, therefore, this study did not require ethical approval from a specific committee.

### 2.2 DNA extractions, PCR and species determination from the hair samples

DNA extractions and pre‐PCR procedures were performed in separate rooms under sterile conditions. Hair and tissue samples were processed independently to avoid contamination with the more concentrated tissue samples. DNA was extracted using the DNeasy Blood and Tissue Kit (QIAGEN). To identify the species of the hair samples, a 564 bp fragment of the mitochondrial D‐loop was amplified by PCR using mustelid‐specific primers and conditions, as previously described (García et al., 2017; Table S3). DNA sequencing of the PCR product allowed the phylogenetic comparison with other sequences from GenBank (Clark et al., 2016), and thus the species determination.

### 2.3 DNA quantification and sex determination by qPCR

Using genomic sequences obtained from tissue samples of the European mink of initial ddRAD libraries (Peterson et al., 2012), species‐specific primers were designed using the Primer3 software (Untergasser et al., 2012) to perform DNA quantification and sex determination of hair samples. Specifically, two primer pairs that amplified a 43 bp autosomal fragment and a 46 bp Y chromosome fragment, respectively, were used (Table S3). DNA was quantified with each primer set in qPCR plates, using three replicates, serial dilutions of a standard sample of known concentration, and a non‐template control, with other conditions as previously described (Escoda et al., 2019). The ratio of Y chromosome to autosomal DNA was used to identify individuals as male (theoretically should be 0.5, but ratios >0.2 and <1.0 were accepted) and female (theoretically 0, but ratios <0.01 were accepted). Samples that fell outside these ranges or with very low autosomal DNA concentrations (<0.1 ng/μL) were considered indeterminate.

### 2.4 ddRAD library preparation and analysis

To obtain genomic sequences, we followed the ddRAD library preparation protocol (Peterson et al., 2012), with some modifications to allow us to process the samples independently (Escoda et al., 2019). Only samples with a DNA concentration >0.5 ng/μL were considered. Between 25 and 200 ng of genomic DNA from each sample was digested with EcoRI and MspI restriction enzymes. After ligation of adapters with specific barcodes for each sample, a fragment of 300–400 base pairs was selected in an E‐Gel electrophoresis system and amplified with three PCR reactions of 20 cycles each. To pool the samples and construct the final libraries, we took a volume of each sample according to its product intensity in an agarose gel, discarding samples that did not amplify sufficiently. The libraries were sequenced on the NextSeq Sequencing System (Illumina) in single‐read runs of 150 bp (Read 1 only) at the Genomics Core Facility of Pompeu Fabra University.

The Stacks 1.48 software (Catchen et al., 2013) was used to assemble the genomic reads of each sample from the different groups of samples, as previously described (Escoda et al., 2019). In all the groups, the following parameters were used for the assembly: minimum number of identical reads required to build a stack *m* = 3; maximum number of differences between alleles of the same individual *M* = 2; and maximum number of differences between alleles of the loci catalog *n* = 2.

The sequences from the tissue samples were used to detect endogenous sequences and thus remove any contaminating DNA present in the hair samples, as previously explained (Escoda et al., 2019). Contamination could come from bacteria or other organisms present on the skin, as well as from other mammalian species that left hairs in the same trap. To filter these sequences, we first assembled the tissue sample reads with Stacks using the parameters described above, exported the sequences to FASTA format, and used them to build a database of endogenous European mink sequences with the Bowtie 2.3.4.3 software (Langmead & Salzberg, 2012). We then filtered the hair sample sequences using Bowtie 2.3.4.3 (Langmead & Salzberg, 2012) with the option “‐‐score‐min L,0,‐0.6”, so that only sequences giving at least one hit to the tissue sample database were retained. A first assembly with *m* = 12 (minimum depth of coverage to call SNPs) and *r* = .51 (minimum proportion of samples) was generated to characterize the assembly quality of the samples. For the group of hair samples that assembled a sufficient number of loci, we performed a new assembly and retrieved SNPs using the Populations program in Stacks with *m* = 12 and *r* = .7. We also removed loci that deviated from the Hardy–Weinberg equilibrium, as assessed with GENEPOP 4.6 (Rousset, 2008).

We generated an additional SNPs dataset from which we removed SNPs with a single variant or singletons, which could be due to erroneous sequences, using the following BCFtools v 1.15 (Li, 2011) command:

*bcftools filter ‐i"MAC>1" $file*

where *$file* is the SNPs file in VCF format.

### 2.5 Analysis of singletons

To evaluate the substitutions associated with the singletons, we collected all singletons with the BCFtools command:

*bcftools filter ‐i"MAC<=1" $file*.

From the resulting file, we applied the following BCFtools command to obtain the substitutions in each sample:

*bcftools query ‐i'GT="alt"' ‐f'[%REF‐%ALT\t%GT\t%SAMPLE\n]' ‐s $sample $file*

where *$sample* is each sample name. From the resulting file (saved in *$file*), substitution classes were counted, and the proportions of each class with respect to the number of SNPs were plotted for each sample.

### 2.6 Sex determination with the ddRAD sequences

To corroborate the sex of the samples, Y‐chromosome sequences were first identified in tissue samples of known males, as previously described (Escoda et al., 2019). These sequences were then used as a database for detecting Y‐chromosome sequences in genomic reads from the hair samples using Bowtie (Langmead & Salzberg, 2012).

### 2.7 Genotyping analysis

Individualization of the hair samples was first performed with the COLONY 2.0.6.6 software (Jones & Wang, 2010), which analyzes all samples simultaneously using a likelihood method. We set up the program parameters with the possibility of detecting duplicates (clones), the full‐likelihood method, inbreeding, medium run length and precision, update of allele frequencies, and an error rate of 0.15 (0.075 for both allelic dropout and false allele rate). The same results were obtained using summed error rates between 0.1 and 0.2, which included the values estimated from the comparison of hair and tissue samples (see below).

The individualization analysis of COLONY was tested using the relatedness coefficient between samples estimated with the program Related 1.0 (Pew et al., 2015). We used the *dyadml* estimator, the three identity‐by‐descent (IBD) states model, and an error rate of 0.15. Sample pairs of the same individual should have a relatedness coefficient close to 1 and a probability of IBD0, also estimated by the program, close to 0.

To determine whether genotyped hair samples coincided with previously live‐trapped specimens, we also used COLONY with a group of samples that included both hair and tissue samples.

### 2.8 Estimation of error rates of the SNPs

For individuals for which we had both hair and tissue samples, we calculated the error rates of the hair samples using the tissue samples as the reference. We separately quantified allelic dropout (e.g., AG to AA changes) and false alleles (e.g., CC to AC changes). Error numbers were divided by the total number of positions compared to calculate the error rates.

### 2.9 Simulations to assess genotyping performance with mixed samples

Due to the nature of the hair‐trapping system, different individuals of European mink could leave hairs on the same adhesive sheet, resulting in samples containing mixed DNA, which could be a major problem for sample individualization. Hair from other species, which can also occur, is filtered out with the European mink tissue sample database and is therefore not a problem for genotyping.

To test the performance of the COLONY software (Jones & Wang, 2010) with mixed samples from different European mink and to find out whether these samples could be incorrectly assigned to new individuals, we bioinformatically generated mixed samples using the reads from six individuals. To do so, we randomly selected a subsample of reads from each individual and concatenated the reads from two individuals in different proportions (10%–90%, 25%–75%, and 50%–50%), creating 15 mixed samples that contained between 1,500,000 and 2,500,000 reads. The artificially mixed samples were added to the group of hair samples, a new Stacks assembly was performed, and COLONY was run using the same parameters as above for the individualization analysis.

### 2.10 Relatedness and inbreeding coefficients

Relatedness among European mink samples was estimated using Related (Pew et al., 2015) with the nine IBD states model, which accounts for inbreeding, and an error rate of 0.15 in the group of hair and tissue samples. Only significant values, as determined with the bootstrap option of Related and 100 replicates, were used. To assess the suitability of the hair samples for obtaining relatedness estimates, we performed a correlation analysis of the significant relatedness coefficients between the hair and tissue samples from individuals for which we had both sample types and the other individuals. In addition, inbreeding coefficients were calculated and compared between the hair and tissue samples from the same individual.

### 2.11 Population size estimates using a SCR model

Studies that integrate non‐invasive genetic data with spatial capture–recapture (SCR) models have greatly improved the estimates of density and population size (Efford, 2004; Kéry et al., 2010; López‐Bao et al., 2018; Srivathsa et al., 2021). We estimated the population size using an SCR model, in which the probability of detection of an individual in a trap is modeled as a function of the distance between its activity center and the trap. We used the R packages SECR 3.2.1 and SECRLINEAR 1.1.1 (Efford, 2019), which allowed us to estimate network distances between traps and activity centers, instead of Euclidean distances, and calculate population densities along a linear habitat such as a river. Network distances have been shown to be highly appropriate for other riverine mammals (Murphy et al., 2021). The model was applied to a shapefile of the river sectors where the hair traps were placed (downloaded from the Ebro River Basin Authority website, http://www.chebro.es/), thus assuming that all optimal habitats were covered by traps. We also used the coordinates of the 127 localities with traps and the capture history of the genotyped individuals, where for each capture we recorded the specimen code, the locality code, and the trap check or occasion (first, second, or third). The traps were considered to be of the “proximity” type, which allows no more than one sample per locality and occasion, but there may be multiple records of a single animal in different traps on each occasion. A half‐normal distribution was used as the detection function, after checking that it best fit the model according to the AIC criterion. Due to the small number of records obtained, we used the simplest model, i.e., without taking heterogeneity into account. Home range statistics were calculated using the RPSV function and the number and confidence interval (CI) of animals were calculated using the region.N function.

## 3 RESULTS

### 3.1 Species identification of hair samples

We genetically analyzed 96 hair samples found in 78 hair traps collected during three trap checks (Table S1 and Figure 2b). After sequencing and analysis of the mitochondrial D‐loop, 76 samples from 30 different localities were found to correspond to European mink (Table S1). The number of positive localities in the three trap checks was 15, 16, and 18, respectively, for a total of 49 positive localities  (Table S1).

Mitochondrial sequencing revealed only two haplotypes of the D‐loop (Table S4), in agreement with the low mitochondrial genetic diversity of the species.

### 3.2 Sex determination of the European mink samples by qPCR

To determine the sex of the European mink samples by qPCR, only samples with more than 0.1 ng/μL of autosomal DNA were considered. The sexed samples belonged to 58 males and three females, indicating a strong sex bias (Table S5).

### 3.3 ddRAD processing

To construct the ddRAD libraries, we selected the best samples in terms of DNA quantity and quality, as determined by qPCR. Thus, we only selected samples with more than 0.5 ng/μL of autosomal DNA and that were correctly sexed by qPCR, which preliminary data showed to be important requirements to increase the chances of a successful ddRAD. When several samples were available from the same locality (i.e., from any of the adhesive sheets and traps at each locality) and occasion, we generally selected a single one, assuming that they all probably came from the same individual. After this selection, a total of 43 samples were included in the ddRAD analysis (Table S5). Of these, 34 samples produced a sufficiently strong PCR product during the library preparation and were further processed and sequenced (Table S5).

An average of 2,798,706 reads per sample was obtained. Filtering with the tissue sample database retained only 36% of these reads as being from European mink, with the remainder being mostly contaminating DNA (Table S6).

A first assembly generated 16,308 loci, but some samples yielded the majority of loci while others assembled <1% (Table S6). We selected 19 samples that contained >1,000,000 assembled reads, equivalent to a depth of coverage of >20×, and rendered >85% of the total loci, as these were the only samples that resulted in unambiguous genotypes and individualization in initial tests. In addition, different *r* values were tested to filter out SNPs present in few samples. We finally selected only those SNPs present in at least 70% of the samples (*r* = .7), which generated 2521 SNPs.

To confirm the sex determinations, we used the Y chromosome sequences obtained by ddRAD. For this analysis, more samples could be used than for genotyping because fewer reads were required. Therefore, we used the 26 samples with more than 100,000 retained reads. The sex determination for these samples was unambiguous (24 were from males and 2 from females; Table S7) and in perfect agreement with the sex determined by qPCR.

### 3.4 Sample individualization

**FIGURE 3:**
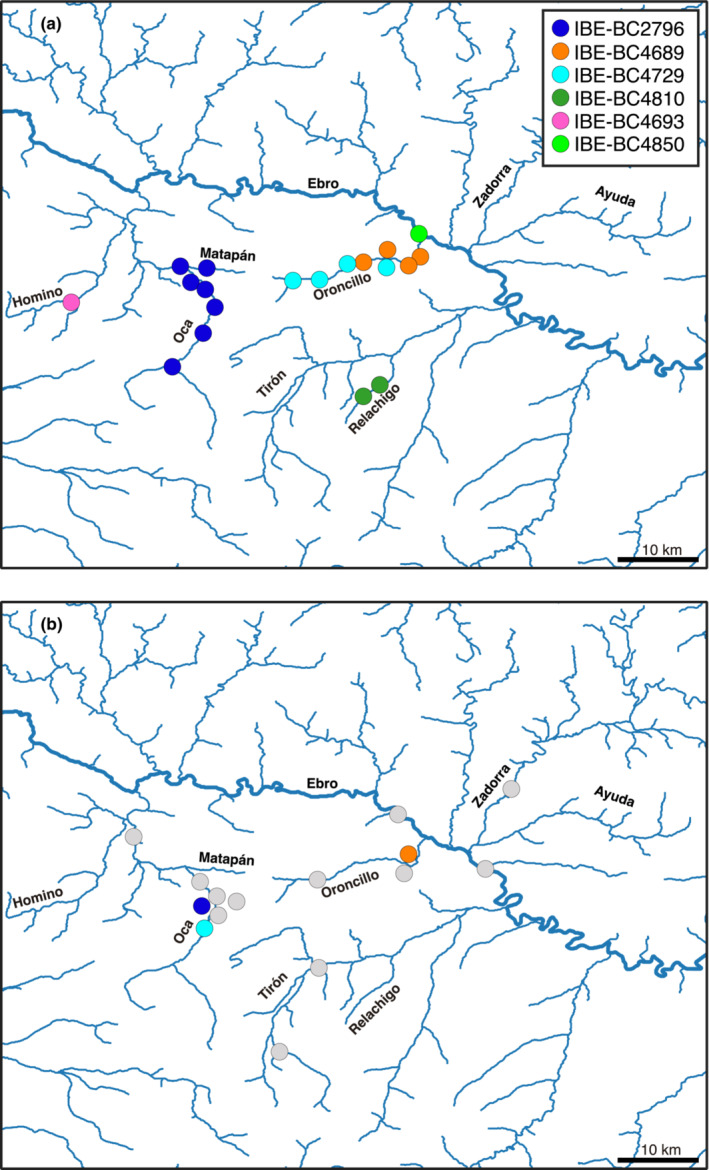
(a) Map of the 19 genotyped hair samples using different colors for the six detected individuals. (b) Map of the live‐trapped European mink showing the corresponding colors of the three individuals detected with hair samples. In both maps, points represent approximate locations of the positive traps.

**FIGURE 4:**
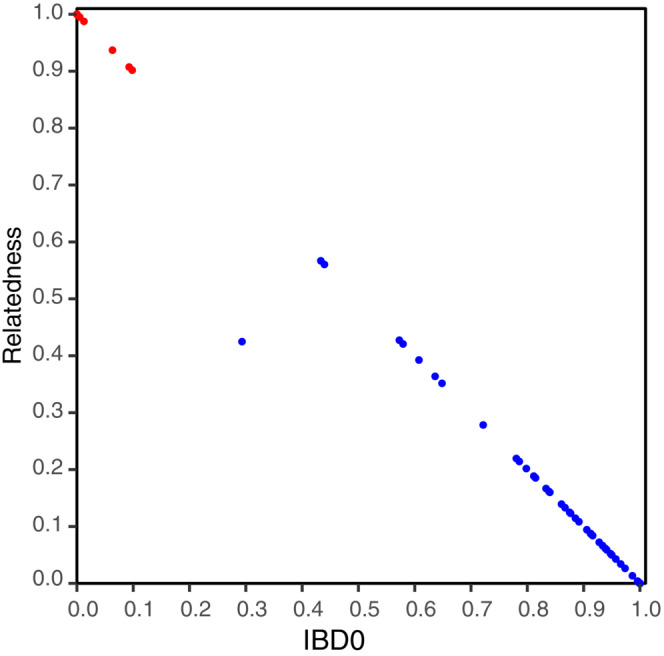
Plot of relatedness coefficients versus IBD0 for each pairwise comparison between hair samples. Comparisons of samples belonging to same individuals inferred by COLONY are represented by red dots whereas blue dots indicate other relationships.

Sample individualization was performed using the SNPs dataset after singleton filtering, which consisted of 753 SNPs and provided more robust results in preliminary analyses than the unfiltered dataset. The COLONY program detected six different individuals from the 19 genotyped hair samples. All sample clusters were detected with a probability score of 1. These individualization results were consistent with sample location, as samples from the same individual tended to be found in the same river (Table S1 and Figure 3a). According to the previous sex determination analyses, the detected individuals corresponded to five males and one female. To assess the individualization analysis, the relatedness and IBD0 values estimated by Related between all sample pairs were plotted (Figure 4). The points corresponding to the comparison of samples from the same individual according to COLONY (in red in Figure 4) were grouped around the expected values.

When tissue and hair samples were compared, we detected three hair samples that matched the genotypes of previously captured individuals (Figure 3b), with a probability score of 1 in the three clusters. The three individuals identified corresponded to males captured between 2018 and 2020.

### 3.5 Estimation of SNP error rates

For the three individuals for which we had both tissue and hair samples, we estimated the genotyping error rates of the hair sequences using the tissue sequences as a reference, with the SNPs after removing singletons (Table S8). The average false allele error rate was 0.05 and the allelic dropout rate was 0.07. Because the error rates were relatively high, we determined the specific substitutions occurring in the hair samples. We observed an excess of C to A changes in most of the samples (Figure S1a). When singletons were removed, most of the C to A changes disappeared (Figure S1b). The same excess of C to A changes was observed when the changes were analyzed in the singletons of the group of hair samples (Figure S1c), indicating that most of the singletons in the hair samples were due to errors and justifying that all the individualization, relatedness, and inbreeding analyses were performed on the SNPs dataset after removing singletons.

### 3.6 Assessment of simulated mixed samples

We used COLONY to perform the individualization of bioinformatically mixed samples. Such mixed samples were inferred to belong to the individual contributing with the highest proportion of reads (75% or 90%) in all cases (Table S9). The three mixed samples containing 50% of the reads from each individual, which were the most difficult to resolve, were grouped with one of the original individuals but, in some cases, remained alone as a new individual. The latter cases are the most problematic because a new individual would be incorrectly inferred. However, the probability of these groups was 0, meaning that they were not reliable and should be discarded (Table S9). This analysis showed that, in principle, the COLONY algorithm is able to satisfactorily perform the individualization analysis, without inferring new individuals even when working with samples that contain a varying proportion of mixed DNA, as long as the probability of the individualization analysis is taken into account.

### 3.7 Population size and home range estimate

**FIGURE 5:**
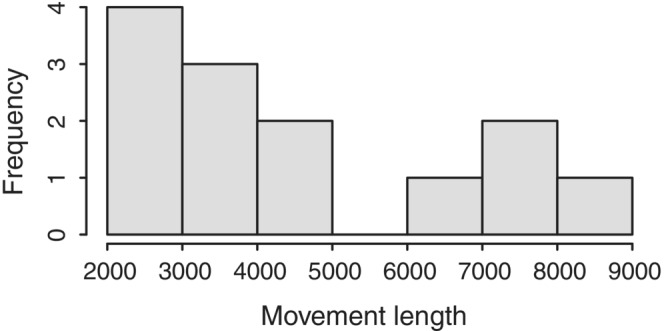
Distribution of movement lengths of detected European mink individuals in hair traps.

Applying an SCR model to the 19 genotyped hair samples from the six individuals resulted in a density of 0.020 individuals/km (95% CI: 0.009–0.045). Considering the total length of the sampled river network (311 km), the estimated population size was 6.2 individuals (95% CI: 6.0–12.2). The maximum dispersal distance was 8.3 km (Figure 5) and the home range was 5.9 km for the 3‐week sampling period, in line with the expected values for the species (Palazón, 2017).

### 3.8 Relatedness and inbreeding coefficients in the population

**FIGURE 6:**
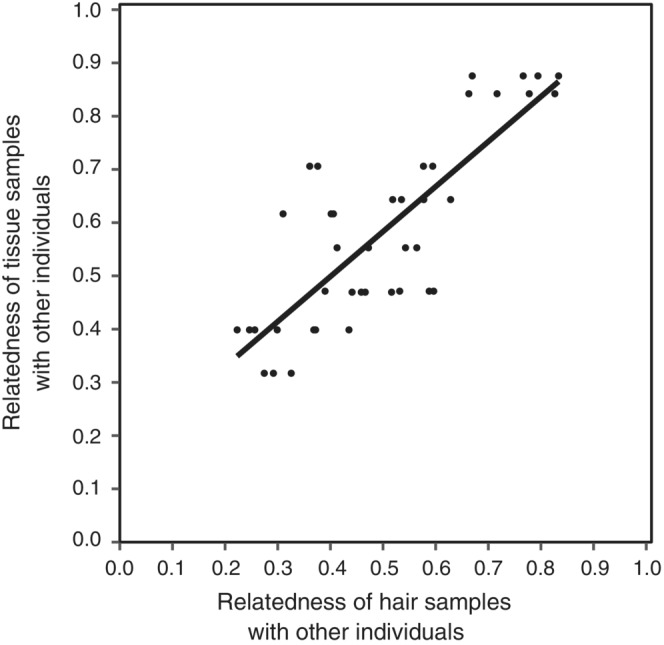
Correlation of significant relatedness coefficients between individuals for which we have both hair and tissue samples, with the other individuals.

By comparing tissue and hair samples from the same individuals, we were able to examine how hair samples performed when estimating kinship relationships. To do this, we compared the significant relatedness coefficients of the hair and tissue replicates of the same individual with the other individuals (Figure 6). We found a Pearson correlation of *r* = .81. Thus, hair samples provided a moderately good approximation of kinship relationships. All types of kinship relationships, from close to more distant, were observed between the individuals in this study (Figure 6). Some of the values were close to 1 due to the use of a model that accounts for inbreeding.

Additionally, for the three individuals for which we had both sample types, the estimated inbreeding coefficients were similar for both hair and tissue samples (Table S10), although those hair samples with a high number of C to A singletons or high genotyping error rates (Figure S1 and Table S8) tended to differ more from the tissue sample. These results indicated that singleton removal was effective for genetic identification and relatedness, but not so much for inbreeding coefficient estimation.

## 4 DISCUSSION

### 4.1 Evaluation of non‐invasive hair tube sampling and its analysis by ddRAD

In this work, we demonstrate the effectiveness of the hair tube technique in detecting the presence of European mink in a population located at the southwestern limit of the species' range, where we obtained 76 hair samples of this species over a 3‐week sampling period. These results suggest that this type of non‐invasive trapping and the genetic identification of the species may be of great value in future studies of the species distribution. In addition, we show that the ddRAD sequences obtained from these samples allowed us to individualize a selection of hair samples and perform relatedness and preliminary inbreeding estimates with them. However, we identified some issues with the DNA from these non‐invasive samples that should be considered to avoid problems in the analyses and interpretation of the results.

#### 4.1.1 Contamination by exogenous sequences

Hair samples can be contaminated by bacteria and other organisms that colonize the hair, as well as by bacteria involved in biodegradation that may have accumulated in the hair prior to collection. In fact, exogenous DNA accounted for the majority of the sequences (Table S6), leaving only 36% of endogenous sequences. Therefore, the exogenous sequences must be filtered using a complete genome of the target species or, in its absence, a database of ddRAD sequences from tissue samples, and only sequences that match such a genome or database should be used. Otherwise, loci from other species may be assembled. To increase the number of samples successfully genotyped, enrichment techniques that increase the proportion of vertebrate DNA relative to bacterial DNA prior to ddRAD library construction may be advantageous (Tyagi et al., 2022).

#### 4.1.2 DNA fragmentation

One of the most important consequences of DNA degradation is fragmentation into small pieces (Briggs et al., 2007). If the fragments are smaller than approximately 300 bp, which is the typical insert size for ddRAD libraries, little or no DNA will be available for sequencing. Samples with a small number of reads can have significant allelic dropout problems. To minimize these problems, it is necessary to remove samples that do not meet certain read count or coverage thresholds, as determined in initial analyses. The allelic dropout rate in this study was high for the SNPs dataset (Table S8), but similar to other studies with non‐invasive samples (Carroll et al., 2018), indicating that the thresholds used were effective.

#### 4.1.3 Oxidative DNA damage

The false allele rate in these hair samples was much higher than in other studies (Carroll et al., 2018). By comparing hair and tissue samples, we observed a high number of C to A changes in most hair samples. Each of these changes was unique and could, therefore, be detected as singleton SNPs. An excess of C to A changes is compatible with DNA oxidation, as previously described for other sample types (Briggs et al., 2007; Chen et al., 2017; Höss et al., 1996; Ma et al., 2019). Oxidation converts guanine to 8‐oxoguanine (G*). During the amplification cycles, G* is misread as a T and pairs with an A instead of a C, causing a C to A change in the complementary chain (Chen et al., 2017; Costello et al., 2013). The fact that the chemical damage occurs on only one of the strands for each damaged site and the way the ddRAD library is prepared (Peterson et al., 2012) results in only the C to A changes being detected by Read 1, but not the complementary G to T changes (Figure S2). To our knowledge, substitutions compatible with DNA oxidation have not been reported in samples used for biodiversity and conservation studies (Carroll et al., 2018). However, this problem has been reported in other research areas, such as ancient DNA (Briggs et al., 2007; Höss et al., 1996), where samples have been shown to undergo oxidation shortly after specimen death, and cancer research (Chen et al., 2017), where the sonication step present in library preparation has been shown to damage samples (Abascal et al., 2021; Costello et al., 2013). Since sonication is not used in ddRAD library preparation, this is not an issue with the ddRAD protocol. In fact, in other published ddRAD studies (e.g., Balmori‐de la Puente et al., 2022; Escoda et al., 2019), samples with an excess of C to A changes are either not found or are very rare and of small magnitude. Rather, the problem in the present work is likely to be related to the preservation of some samples. Many different agents can directly or indirectly cause DNA oxidation (Cadet & Davies, 2017; Lindahl, 1993). In the case of the hair samples from hair traps, the glue used in the adhesive sheets may have contributed to oxidation, but other agents cannot be excluded. Oxidation does not necessarily cause DNA fragmentation or affect PCR efficiency (Höss et al., 1996), so oxidized samples may generate many reads, giving the false impression that they are in good condition and potentially leading to erroneous interpretations. Therefore, an analysis of the excess of C to A changes is essential to detect oxidized samples and take the necessary steps to avoid artifactual results.

#### 4.1.4 Sample mixing

Hair traps may contain hair from more than one European mink individual, which can also lead to genotyping errors. We have shown that the COLONY program (Jones & Wang, 2010) used for individualization is effective in genetically characterizing bioinformatically mixed samples from two individuals when one of them is dominant. We could not determine whether mixing occurred in the hair samples collected here, but some of the samples with high genotyping errors may have been affected by this circumstance.

By solving or mitigating these technical difficulties, we were able to reliably genotype and genomically analyze 19 hair samples, representing 25% of the 76 European mink samples. Because up to four different samples could be collected at each of the 49 positive localities, the locality success rate was higher (39%). However, the proportion of genotypes called was lower than in other studies using SNP panels (Fitak et al., 2016; von Thaden et al., 2017). Therefore, these non‐invasive samples represented a challenging case study for ddRAD genotyping, but they allowed us to make progress toward a better understanding of the processes that may affect them. An important implication of these results is that precautions during sampling and sample preservation are more important with genomic techniques than with traditional genetic techniques. In this work, hair samples were collected at 7‐day intervals, which may have resulted in some samples being exposed to the environment and chemicals in the adhesive sheet for too long, and increases the risk of collecting mixed samples from different individuals. In the future, samples should be collected more frequently (e.g., just at 2‐ or 3‐day intervals), which may be especially important in areas with higher densities of the species. In addition, different types of adhesive sheets should be tested to ensure that they do not promote oxidation or other types of chemical degradation of the DNA. These precautions should result in a higher success rate of genotyped and genomically characterized samples.

### 4.2 Application of non‐invasive sampling and genomic techniques to study the population size and genetic parameters of the European mink

Despite the challenges in analyzing the samples, the hair traps proved to be an effective method for detecting individuals in a low‐density area, such as the peripheral area studied here, making this technique very useful for monitoring the presence of the species in the most problematic areas for its conservation. However, the number of samples and individuals identified (19 and 6, respectively; Figure 3a) was too low to apply the most advanced SCR models, so we could only apply a simple model that did not account for different sources of heterogeneity, which may have led to underestimation. Nevertheless, the results showed a very low density, confirming the small population size of the European mink in this peripheral area.

A strong sex ratio bias was also observed, with five males and one female. Previous live trapping in the area showed a similar trend, as only 22% of the individuals detected in recent years (2018–2020) were females. An underrepresentation of females in hair traps has also been noted in other studies of mustelids (Croose et al., 2019). This low sex ratio could be due to a more cautious behavior of females toward the traps, which would be a limitation of the method. Nevertheless, it cannot be excluded that the natural sex ratio is altered at the edge of the species' distribution due to the higher dispersal activity of males, particularly during the reproductive season (Palazón, 2017). Additional surveys should be conducted in different time periods to obtain more hair samples and independently estimate the detectability and population size for males and females, and thus better follow the demographic evolution of this peripheral population.

In addition to individualization, we demonstrated the potential of the ddRAD sequences to estimate relatedness between individuals and inbreeding. The number of genotyped individuals was small to reconstruct kinship networks, but many kinship relationships of different degrees could be observed (Figure 4), suggesting that there is good intrapopulation connectivity in the area (Escoda et al., 2019). The inbreeding coefficient, one of the most critical genetic indicators for individuals and populations (Leroy et al., 2018), could also be determined from the hair samples with the fewest genotyping errors. Despite the peripheral nature of this population, the inbreeding coefficients were variable, with some of the analyzed individuals basically not inbred at all, while others were more inbred (Table S10). However, it is well known that the overall genetic diversity of the European mink is low (Cabria et al., 2015; Skorupski, 2020), and therefore a larger study analyzing the important genetic parameters of the species is essential.

### 4.3 Implications of the results for the conservation of the European mink and other endangered mammals

The hair trapping system and the genetic identification of hair samples offer several advantages for the genetic monitoring and management of an endangered species such as the European mink, especially compared to traditional trapping systems. As we have shown, a good approximation of the distribution of the species can be obtained. In addition, this type of trapping system allows the detection of individuals in low‐density areas, such as the one analyzed here, located at the edge of the species' distribution. Furthermore, this can be done systematically in different populations and without excessive logistical or economic difficulties. Finally, and most importantly, all these data can be obtained without disturbing the animals and putting them at risk.

We have also demonstrated the potential of ddRAD to perform individualization, relatedness, inbreeding, and connectivity analyses using non‐invasively obtained hair samples. However, the high genotyping error rates found in some samples mean that several steps of this monitoring method still need to be optimized. For example, different adhesive sheets and DNA preservation methods should be tested to avoid chemical degradation of the samples. Also, hair samples from the same sheet fixed at different positions may be separated to minimize the possibility of sample mixing from different individuals. In addition, samples should be collected in shorter periods of time, which would allow extraction of higher quality DNA and also help to avoid mixing of samples. This should not imply a much greater sampling effort for species that come into the trap early, as appears to be the case for the European mink, although pilot surveys may be necessary to improve the sampling conditions. Despite all the additional effort required, genomics‐based non‐invasive monitoring methods have enormous potential to provide information of great value for species conservation and management, so it is of the utmost importance that these technologies continue to be developed and improved.

## AUTHOR CONTRIBUTIONS

**Alfonso Balmori‐de la Puente:** Conceptualization (equal); investigation (equal); methodology (equal); writing – original draft (equal); writing – review and editing (equal). **Lídia Escoda:** Investigation (equal); methodology (equal); writing – review and editing (equal). **Ángel Fernández‐González:** Conceptualization (equal); funding acquisition (equal); investigation (equal); methodology (equal); supervision (equal); writing – review and editing (equal). **Daniel Menéndez‐Pérez:** Investigation (equal); methodology (equal); writing – review and editing (equal). **Jorge González‐Esteban:** Conceptualization (equal); investigation (equal); methodology (equal); writing – review and editing (equal). **Jose Castresana:** Conceptualization (equal); funding acquisition (equal); investigation (equal); methodology (equal); supervision (equal); writing – original draft (equal); writing – review and editing (equal).

## CONFLICT OF INTEREST STATEMENT

None declared.

### OPEN RESEARCH BADGES

This article has earned an Open Data badge for making publicly available the digitally‐shareable data necessary to reproduce the reported results. The data is available at [https://doi.org/10.5061/dryad.np5hqbzz2].

## Supporting information


Appendix S1

## Data Availability

The ddRAD sequences are available from Dryad: https://doi.org/10.5061/dryad.np5hqbzz2. The D‐loop sequences are available from GenBank: accession numbers OQ383427 and OQ383428.
