# Supplementary material for: Evaluating the use of non‐invasive hair sampling and ddRAD to characterize populations of endangered species: Application to a peripheral population of the European mink
Source: Ecol Evol. 2023 Sep 18;13(9):e10530. doi: 10.1002/ece3.10530 (PMC10506391; doi:10.1002/ece3.10530)
Supplement: Supplementary file 1 — Appendix S1 [file ECE3-13-e10530-s001.pdf]

Supporting Information

Evaluating the use of non-invasive hair sampling and ddRAD to characterize populations of endangered species: application to a peripheral population of the European mink

Alfonso Balmori-de la Puente [1], Lídia Escoda [1], Ángel Fernández-González [2], Daniel Menéndez-Pérez [2], Jorge González-Esteban [3], Jose Castresana [1]

[1] Institute of Evolutionary Biology (CSIC-Universitat Pompeu Fabra), Barcelona, Spain

[2] Biosfera Consultoría Medioambiental S.L., Oviedo, Spain

[3] DESMA Estudios Ambientales, Ituren (Navarra), Spain

Correspondence: Jose Castresana, Institute of Evolutionary Biology (CSIC-Universitat Pompeu Fabra), Passeig Marítim de la Barceloneta 37, 08003 Barcelona, Spain

Email: jose.castresana@csic.es

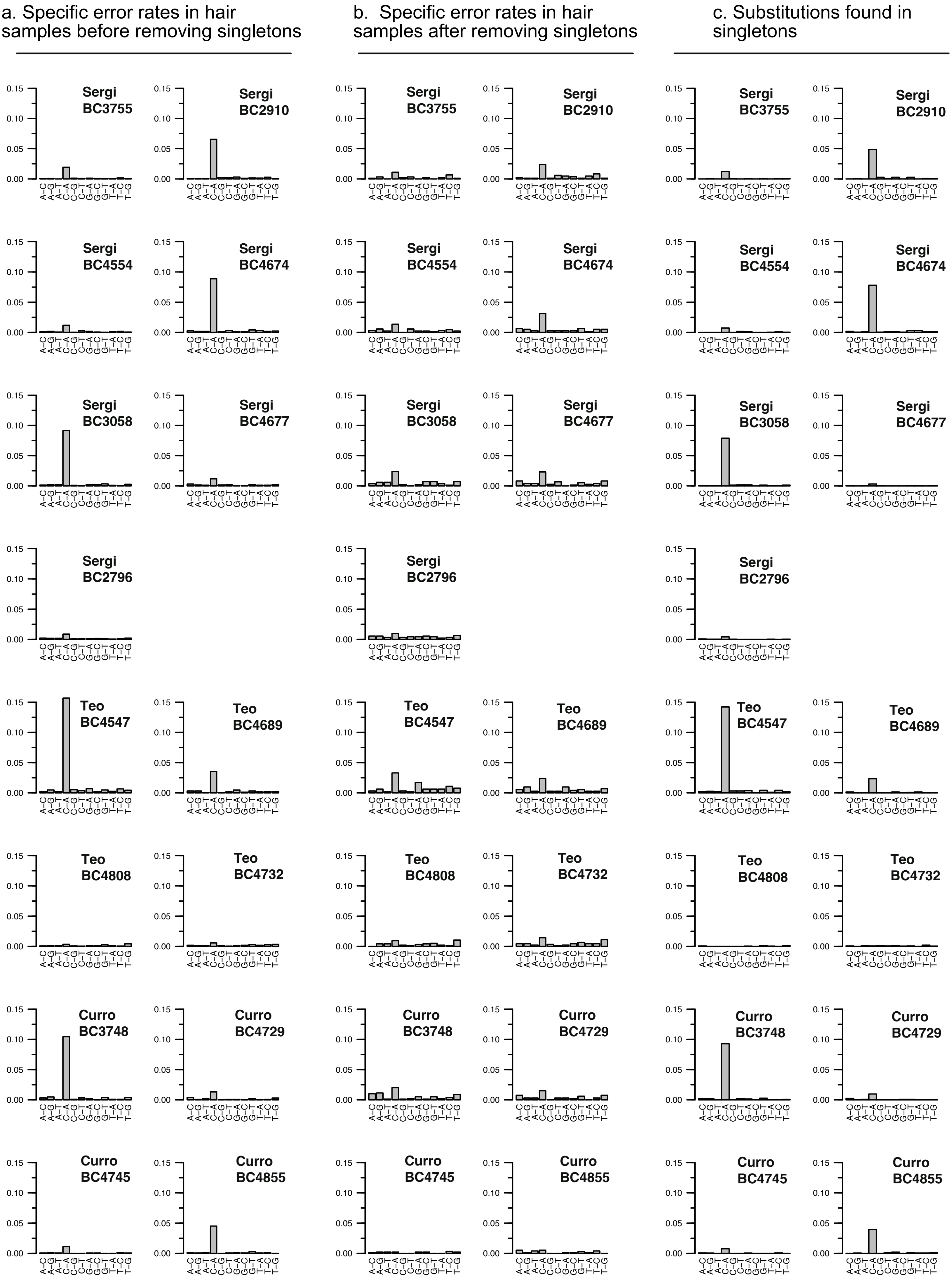

Figure S1. Barplots showing the proportion of nucleotide substitutions found in the comparison of hair and tissue samples from the same individual (a) before and (b) after removing singletons. (c) Proportion of changes associated to the singletons in the group of the hair samples.

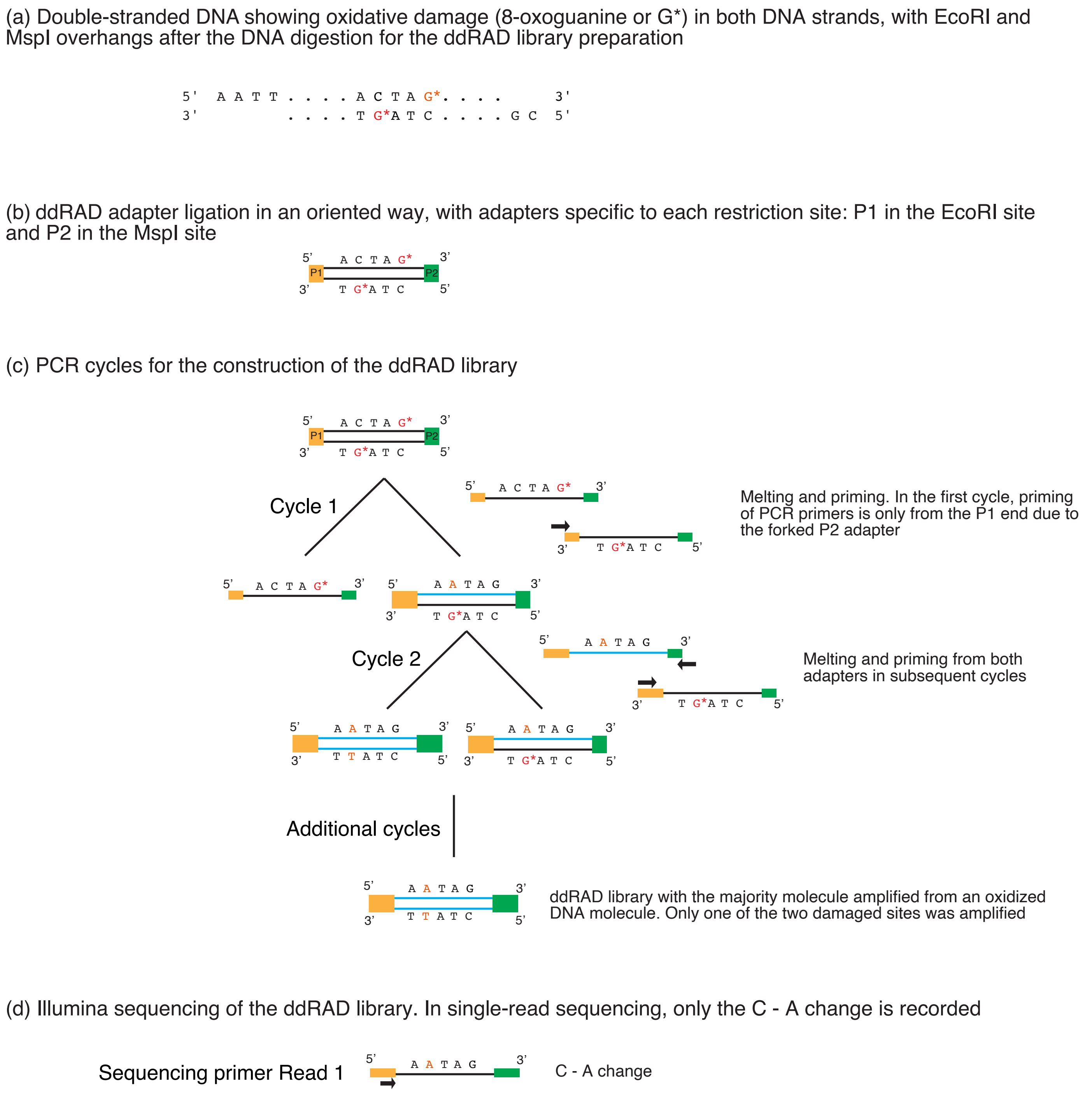

Figure S2. Diagram showing the preparation of a ddRAD library of DNA with oxidative damage and a possible mechanism of how oxidative damage, which generates 8-oxoguanine (G*) from guanine, affects the sequencing of the ddRAD library. (a) In this example, there are two damaged bases, one on each DNA strand. (b) Adapters P1 (with the Read 1 primer sequence being attached to the 5' end of the top strand) and P2 (with Read 2 in corresponding position) are ligated in an oriented way. (c) During the PCR cycles, only the top DNA strand is amplified as a result of the forked P2 adapter (Peterson et al. 2012). (d) During sequencing, this results in only the G* present on the bottom strand being reflected in a C-A change in Read 1 sequences, while the G* present on the top strand is not detected by this read. The effect of G* in ddRAD, producing only C-A changes, is opposite to that observed in typical Illumina libraries with oxidized bases, where Read 1 reflects only G-T changes due to the different orientation of the sequencing primers in the library (Chen et al. 2017).

Table S1. Hair samples analyzed in this study with information about locality data, position of the two traps at each locality (named as "U": upper and "L": lower), adhesive sheet (called "A" and "B" sheets in case of analyzing the two sheets in one trap, otherwise there is only "A" sheet), revision, and species determination.

**Sample Code**	**Locality**	**Position-Sheet**	**Revision**	**Species**
BC3768	Zorita_TP002	U-A	First	Not determined
BC3765	Oca_TP003	L-A	First	*Martes foina*
BC3767	Oca_TP003	L-B	First	*Martes foina*
BC4604	Oca_TP008	L-A	First	*Mustela lutreola*
BC3755	Oca_TP008	L-B	First	*Mustela lutreola*
BC4658	Oca_TP009	U-A	First	*Mustela lutreola*
BC3756	Oca_TP009	U-B	First	*Mustela lutreola*
BC4657	Oca_TP011	L-A	First	*Mustela lutreola*
BC4667	Oca_TP011	L-B	First	*Mustela lutreola*
BC2910	Oca_TP012	L-A	First	*Mustela lutreola*
BC4551	Oca_TP012	L-B	First	*Mustela lutreola*
BC3741	Oca_TP013	U-A	First	*Mustela lutreola*
BC3724	Oca_TP014	L-A	First	*Mustela lutreola*
BC4571	Oca_TP015	L-A	First	*Mustela lutreola*
BC4555	Oca_TP016	U-A	First	*Mustela lutreola*
BC4669	Oca_TP016	U-B	First	*Mustela lutreola*
BC4554	Oca_TP017	L-A	First	*Mustela lutreola*
BC4664	Oca_TP017	L-B	First	*Mustela lutreola*
BC4659	Oca_TP017	U-A	First	*Mustela lutreola*
BC4656	Oca_TP017	U-B	First	*Mustela lutreola*
BC3723	Oca_TP019	L-A	First	*Mustela lutreola*
BC3747	Oca_TP030	L-A	First	*Martes foina*
BC3730	Oca_TP030	L-B	First	*Martes foina*
BC3735	Bañuelos_TP003	U-A	First	*Mustela nivalis*
BC4547	Oroncillo_TP006	L-A	First	*Mustela lutreola*
BC4574	Oroncillo_TP006	U-A	First	*Mustela lutreola*
BC3727	Oroncillo_TP007	L-A	First	*Mustela lutreola*
BC3748	Oroncillo_TP007	U-A	First	*Mustela lutreola*
BC3740	Oroncillo_TP008	L-A	First	*Mustela lutreola*
BC3745	Oroncillo_TP008	U-A	First	*Mustela lutreola*
BC3771	Oroncillo_TP008	U-B	First	*Mustela lutreola*
BC3763	Oroncillo_TP009	L-A	First	*Mustela lutreola*
BC3762	Redecilla_TP001	U-A	First	Not determined
BC3734	Redecilla_TP001	U-B	First	Not determined
BC3764	Relachigo_TP002	L-A	First	*Mustela lutreola*
BC3759	Relachigo_TP002	U-A	First	*Mustela lutreola*
BC3751	Relachigo_TP002	U-B	First	*Mustela lutreola*
BC4693	Homino_TP002	U-A	Second	*Mustela lutreola*
BC4738	Homino_TP005	U-A	Second	Not determined
BC3773	Homino_TP008	L-A	Second	*Martes foina*
BC3113	Zorita_TP002	U-A	Second	Not determined
BC4730	Zorita_TP002	U-B	Second	Not determined
BC4672	Oca_TP004	U-A	Second	*Martes foina*
BC4727	Oca_TP014	L-A	Second	*Mustela lutreola*
BC4692	Oca_TP014	L-B	Second	Not determined
BC4674	Oca_TP015	U-A	Second	*Mustela lutreola*
BC3276	Oca_TP016	U-A	Second	*Mustela lutreola*
BC4699	Oca_TP017	U-A	Second	*Mustela lutreola*
BC3058	Oca_TP018	L-A	Second	*Mustela lutreola*
BC4694	Oca_TP019	U-A	Second	*Mustela lutreola*
BC4677	Oca_TP020	U-A	Second	*Mustela lutreola*
BC4688	Matapán_TP002	L-A	Second	*Mustela lutreola*
BC2796	Matapán_TP002	U-A	Second	*Mustela lutreola*
BC4729	Oroncillo_TP005	U-A	Second	*Mustela lutreola*
BC4681	Oroncillo_TP006	U-A	Second	*Mustela lutreola*
BC4723	Oroncillo_TP007	L-A	Second	*Mustela lutreola*
BC3048	Oroncillo_TP007	U-A	Second	*Mustela lutreola*
BC3035	Oroncillo_TP008	L-A	Second	*Mustela lutreola*
BC4689	Oroncillo_TP009	L-A	Second	*Mustela lutreola*
BC4775	Oroncillo_TP009	U-B	Second	*Mustela lutreola*
BC3037	Oroncillo_TP011	L-A	Second	*Mustela lutreola*
BC1449	Oroncillo_TP011	L-B	Second	*Mustela lutreola*
BC2463	Ebro_TP007	L-A	Second	*Mustela lutreola*
BC4746	Zadorra_TP002	U-A	Second	Not determined
BC4767	Homino_TP008	U-B	Third	Not determined
BC4768	Homino_TP009	L-A	Third	*Mustela lutreola*
BC4782	Castil_TP001	L-A	Third	*Mustela lutreola*
BC4759	Zorita_TP002	L-A	Third	Not determined
BC4744	Zorita_TP002	U-A	Third	Not determined

BC4853	Oca_TP001	U-A	Third	*Mustela lutreola*
BC4803	Oca_TP001	U-B	Third	*Mustela lutreola*
BC4780	Oca_TP016	U-A	Third	Not determined
BC4840	Oca_TP017	L-A	Third	*Mustela lutreola*
BC4832	Oca_TP017	U-A	Third	*Mustela lutreola*
BC4771	Oca_TP017	U-B	Third	*Mustela lutreola*
BC4713	Oca_TP018	U-A	Third	*Mustela lutreola*
BC4783	Oca_TP019	L-A	Third	*Mustela lutreola*
BC4756	Oca_TP019	U-A	Third	*Mustela lutreola*
BC4829	Oroncillo_TP001	L-A	Third	*Mustela lutreola*
BC4745	Oroncillo_TP001	U-A	Third	*Mustela lutreola*
BC4855	Oroncillo_TP002	L-A	Third	*Mustela lutreola*
BC4774	Oroncillo_TP003	U-A	Third	*Mustela lutreola*
BC4823	Oroncillo_TP004	L-A	Third	*Mustela lutreola*
BC4817	Oroncillo_TP005	U-A	Third	*Mustela lutreola*
BC4848	Oroncillo_TP006	U-A	Third	*Mustela lutreola*
BC4808	Oroncillo_TP007	L-A	Third	*Mustela lutreola*
BC4852	Oroncillo_TP007	U-A	Third	*Mustela lutreola*
BC4804	Oroncillo_TP008	L-A	Third	*Mustela lutreola*
BC4732	Oroncillo_TP008	U-A	Third	*Mustela lutreola*
BC4818	Oroncillo_TP008	U-B	Third	*Mustela lutreola*
BC4784	Oroncillo_TP009	L-A	Third	*Mustela lutreola*
BC4850	Oroncillo_TP011	U-A	Third	*Mustela lutreola*
BC4325	Tirón_TP016	U-A	Third	Not determined
BC4810	Relachigo_TP001	U-A	Third	*Mustela lutreola*
BC4793	Relachigo_TP002	L-A	Third	*Mustela lutreola*
BC4317	Relachigo_TP002	U-A	Third	*Mustela lutreola*

Table S2. Tissue samples analyzed in this study with information about locality data, collection date (in format dd/mm/yyyy) and sex of European mink obtained from live trapping. Names of captured specimens detected with hair traps are given in parenthesis after the specimen code. The specimen code consists of the sample code of one of the samples from that specimen, adding "IBE-" as prefix.

Specimen code	Sample code	Locality	Collection date	Sex
IBE-BC3411	BC3411	Oca	02/11/2017	Female
IBE-BC3429	BC3429	Oroncillo	14/11/2017	Female
IBE-BC3388	BC3388	Tirón	12/03/2017	Female
IBE-BC3384	BC3384	Tirón	27/03/2017	Female
IBE-BC3399	BC3399	Ebro	31/10/2017	Female
IBE-BC3460	BC3460	Zadorra	23/02/2017	Male
IBE-BC4729 (Curro)	BC3179	Oca	18/11/2018	Male
IBE-BC3458	BC3458	Oca	01/10/2018	Male
IBE-BC3407	BC3407	Oca	05/01/2018	Female
IBE-BC2796 (Sergi)	BC3939	Oca	25/10/2019	Male
IBE-BC3973	BC3973	Oca	26/10/2019	Female
IBE-BC3717	BC3717	Oca	11/12/2019	Male
IBE-BC3709	BC3709	Oroncillo	26/11/2019	Male
IBE-BC4689 (Teo)	BC4012	Oroncillo	21/01/2020	Male
IBE-BC4310	BC4310	Ebro	07/02/2020	Male

Table S3. Mitochondrial primers used in this study for species determination by PCR (primer with '*' was used without modification from García et al. 2017 and the other was slightly modified) and primers used for amplification of an autosomal and a Y chromosome fragment by qPCR of the European mink. The fragment length is given without counting the primers.

Name	Sequence 5' to 3'	Fragment	Length (bp)
Mink_D-loop-F	ATCAGCACCCAAAGCTGAYATTCTA	Mitochondrial	564
Mink_D-loop-R*	TGTGTATGTCCTGTGACCATTGACT	Mitochondrial	564
Mink_115036_aut-F	CCTCCTAACTCTATTTTAAGACAAGCA	Autosomal	43
Mink_115036_aut-R	TCCTACCCAGACCAAGCAAC	Autosomal	43
Mink_98899_Y-F	ATGGCAGTAATGGACCTCGT	Y chromosome	46
Mink_98899_Y-R	CCCCCTAACCATATTTCCAA	Y chromosome	46

Table S4. D-loop haplotypes of the European mink samples. They are identical except that haplotype 2 has a gap in the middle of the sequence.

Sample Code	D-loop haplotype	Sample Code	D-loop haplotype
BC4604	1	BC2796	1
BC3755	1	BC4729	2
BC4658	1	BC4681	2
BC3756	1	BC4723	2
BC4657	1	BC3048	2
BC4667	1	BC3035	1
BC2910	1	BC4689	1
BC4551	1	BC4775	1
BC3741	1	BC3037	1
BC3724	1	BC1449	1
BC4571	1	BC2463	1
BC4555	1	BC4768	1
BC4669	1	BC4782	1
BC4554	1	BC4853	2
BC4664	1	BC4803	2
BC4659	1	BC4840	1
BC4656	1	BC4832	1
BC3723	1	BC4771	1
BC4547	1	BC4713	1
BC4574	2	BC4783	1
BC3727	2	BC4756	1
BC3748	2	BC4829	2
BC3740	1	BC4745	2
BC3745	2	BC4855	2
BC3771	2	BC4774	2
BC3763	1	BC4823	2
BC3764	1	BC4817	2
BC3759	1	BC4848	2
BC3751	1	BC4808	1
BC4693	1	BC4852	1
BC4727	1	BC4804	1
BC4674	1	BC4732	1
BC3276	1	BC4818	1
BC4699	1	BC4784	1
BC3058	1	BC4850	2
BC4694	1	BC4810	1
BC4677	1	BC4793	1
BC4688	1	BC4317	1

Table S5. DNA concentration by qPCR, sex determination of 76 European mink hair samples and criteria for selecting samples for ddRAD. The concentration of autosomal and Y chromosome DNA is given in ng/µl. Samples included in the ddRAD libraries are marked with X. Samples that did not amplify during library preparation are denoted as (X). The specimen code represents genotyping results of correctly genotyped samples. Names of the previously captured specimens are given in parenthesis after the specimen code.

Sample Code	Specimen Code of genotyped samples	[Autosomal DNA]	[Y DNA]	[Y DNA] / [Autosomal DNA]	Sex	Criteria for ddRAD selection
BC4604		8.14	4.16	0.51	Male	X
BC3755	IBE-BC2796 (Sergi)	4.83	2.18	0.45	Male	X
BC4658		0.57	0.36	0.62	Male	X
BC3756		0.47	0.46	0.98	Male	Discarded: Low DNA
BC4657		0.12	0.09	0.81	Male	Discarded: Low DNA
BC4667		0.10	0.07	0.70	Male	Discarded: Low DNA
BC2910	IBE-BC2796 (Sergi)	0.69	0.21	0.31	Male	X
BC4551		0.06	0.01		Very low DNA	Discarded: Very low DNA
BC3741		0.00	0.02		Very low DNA	Discarded: Very low DNA
BC3724		13.77	6.81	0.49	Male	X
BC4571		2.34	1.33	0.57	Male	X
BC4555		0.17	0.06	0.36	Male	Discarded: Low DNA
BC4669		0.16	0.05	0.28	Male	Discarded: Low DNA
BC4554	IBE-BC2796 (Sergi)	0.97	0.35	0.36	Male	X
BC4664		0.00	0.00		Very low DNA	Discarded: Very low DNA
BC4659		0.74	0.22	0.29	Male	Discarded: Repeated locality
BC4656		0.29	0.42	1.45	High ratio	Discarded: Low DNA
BC3723		1.28	0.81	0.63	Male	(X)
BC4547	IBE-BC4689 (Teo)	2.85	1.47	0.52	Male	X
BC4574		2.34	0.76	0.32	Male	Discarded: Repeated locality
BC3727		3.69	2.32	0.63	Male	Discarded: Repeated locality
BC3748	IBE-BC4729 (Curro)	11.53	6.27	0.54	Male	X
BC3740		9.62	4.99	0.52	Male	X
BC3745		0.63	0.00	0.00	Female	X
BC3771		4.81	0.00	0.00	Female	X
BC3763		2.58	1.03	0.40	Male	(X)
BC3764		1.86	1.52	0.82	Male	X
BC3759		4.46	1.81	0.41	Male	X
BC3751	IBE-BC4810	0.59	0.16	0.27	Male	X
BC4693	IBE-BC4693	4.20	2.55	0.61	Male	X
BC4727		0.00	0.00		Very low DNA	Discarded: Very low DNA
BC4674	IBE-BC2796 (Sergi)	0.87	0.86	0.98	Male	X
BC3276		0.04	0.08		Very low DNA	Discarded: Very low DNA
BC4699		0.95	1.24	1.31	High ratio	Discarded: High ratio
BC3058	IBE-BC2796 (Sergi)	0.65	0.34	0.52	Male	X
BC4694		0.58	0.24	0.41	Male	X
BC4677	IBE-BC2796 (Sergi)	2.33	1.00	0.43	Male	X
BC4688		0.96	0.45	0.47	Male	Discarded: Repeated locality
BC2796	IBE-BC2796 (Sergi)	3.96	2.74	0.69	Male	X
BC4729	IBE-BC4729 (Curro)	18.00	9.80	0.54	Male	X
BC4681		29.57	30.42	1.03	High ratio	Discarded: High ratio
BC4723		2.99	4.19	1.40	High ratio	Discarded: High ratio
BC3048		1.55	1.33	0.86	Male	X
BC3035		1.25	0.97	0.78	Male	(X)
BC4689	IBE-BC4689 (Teo)	2.67	1.85	0.69	Male	X
BC4775		0.00	0.02		Very low DNA	Discarded: Very low DNA
BC3037		0.11	0.27	2.38	High ratio	Discarded: Low DNA
BC1449		1.05	0.36	0.34	Male	(X)
BC2463		0.44	0.35	0.78	Male	Discarded: Low DNA
BC4768		0.23	0.16	0.71	Male	Discarded: Low DNA
BC4782		0.63	0.23	0.37	Male	(X)
BC4853		1.48	0.90	0.61	Male	(X)
BC4803		0.06	0.12		Very low DNA	Discarded: Very low DNA
BC4840		1.95	0.93	0.48	Male	X
BC4832		0.39	0.18	0.46	Male	Discarded: Low DNA
BC4771		0.40	0.19	0.49	Male	Discarded: Low DNA
BC4713		0.00	0.03		Very low DNA	Discarded: Very low DNA

BC4783		0.60	0.16	0.27	Male	Discarded: Repeated locality
BC4756		4.23	1.32	0.31	Male	(X)
BC4829		3.88	1.26	0.32	Male	Discarded: Repeated locality
BC4745	IBE-BC4729 (Curro)	10.82	2.40	0.22	Male	X
BC4855	IBE-BC4729 (Curro)	2.16	0.72	0.33	Male	X
BC4774		1.68	1.35	0.80	Male	X
BC4823		0.81	0.48	0.58	Male	X
BC4817		12.10	4.38	0.36	Male	X
BC4848		6.92	2.28	0.33	Male	(X)
BC4808	IBE-BC4689 (Teo)	1.45	0.79	0.54	Male	X
BC4852		4.46	1.14	0.26	Male	Discarded: Repeated locality
BC4804		2.54	0.76	0.30	Male	Discarded: Repeated locality
BC4732	IBE-BC4689 (Teo)	1.15	0.56	0.49	Male	X
BC4818		0.15	0.09	0.58	Male	Discarded: Low DNA
BC4784		7.94	2.66	0.33	Male	(X)
BC4850	IBE-BC4850	21.13	0.00	0.00	Female	X
BC4810	IBE-BC4810	13.20	4.81	0.36	Male	X
BC4793		0.93	0.14	0.15	Low ratio	Discarded: Low ratio
BC4317		0.67	0.05	0.07	Low ratio	Discarded: Low ratio

Table S6. Basic statistics of the bioinformatic analyses of the ddRAD libraries of the group of 34 hair samples before and after filtering with reads from tissue samples to discard exogenous sequences.

Sample Code	Specimen code after genotyping	Sequenced reads	Retained reads belonging to Europen mink	% Retained reads bowtie	Assembled reads	Assembled loci	Coverage filtered	Assembled loci	% loci
BC4604		999,287	82,802	8.3	72,089	9,324	7.7	531	3
BC3755	IBE-BC2796	7,066,367	4,406,140	62.4	4,220,649	63,343	66.6	16,049	98
BC4658		793,691	160,192	20.2	152,238	9,199	16.5	3,550	22
BC2910	IBE-BC2796	3,701,977	1,880,090	50.8	1,785,069	54,440	32.8	15,666	96
BC3724		916,987	323,697	35.3	302,757	25,002	12.1	5,290	32
BC4571		1,174,329	543,293	46.3	517,214	35,468	14.6	9,640	59
BC4554	IBE-BC2796	3,918,480	2,775,327	70.8	2,639,451	55,912	47.2	15,736	96
BC4547	IBE-BC4689	2,530,311	1,336,689	52.8	1,274,205	49,374	25.8	14,203	87
BC3748	IBE-BC4729	2,551,832	1,722,327	67.5	1,665,620	61,366	27.1	15,602	96
BC3740		245,304	54,504	22.2	47,052	6,297	7.5	244	1
BC3745		1,039,623	99,211	9.5	93,621	8,444	11.1	1,631	10
BC3771		1,991,584	286,741	14.4	272,463	19,025	14.3	5,155	32
BC3764		460,047	12,143	2.6	9,948	1,047	9.5	87	1
BC3759		1,164,959	353,400	30.3	330,538	28,799	11.5	6,403	39
BC3751	IBE-BC4810	5,018,036	2,724,128	54.3	2,597,215	58,598	44.3	15,667	96
BC4693	IBE-BC4693	3,615,291	2,473,441	68.4	2,392,279	62,225	38.4	16,045	98
BC4674	IBE-BC2796	3,919,032	1,506,203	38.4	1,439,836	57,753	24.9	15,219	93
BC3058	IBE-BC2796	2,607,184	1,722,164	66.1	1,667,385	58,587	28.5	15,623	96
BC4694		3,217,482	476,076	14.8	451,167	30,277	14.9	8,814	54
BC4677	IBE-BC2796	4,417,565	1,320,049	29.9	1,241,618	56,514	22.0	15,882	97
BC2796	IBE-BC2796	5,261,285	2,477,389	47.1	2,390,942	65,949	36.3	16,197	99
BC4729	IBE-BC4729	3,342,918	1,212,466	36.3	1,151,014	55,573	20.7	14,427	88
BC3048		533,177	133,189	25.0	121,635	12,400	9.8	2,078	13
BC4689	IBE-BC4689	3,388,682	1,497,808	44.2	1,426,396	58,662	24.3	15,352	94
BC4840		127,781	12,599	9.9	11,352	1,084	10.5	55	0
BC4745	IBE-BC4729	7,792,637	4,479,618	57.5	4,252,287	79,148	53.7	16,070	99
BC4855	IBE-BC4729	3,372,681	1,701,972	50.5	1,607,897	62,483	25.7	14,457	89
BC4774		1,111,175	33,857	3.0	29,852	3,407	8.8	147	1
BC4823		515,726	23,378	4.5	21,108	1,674	12.6	71	0
BC4817		435,164	25,982	6.0	19,141	2,947	6.5	70	0
BC4808	IBE-BC4689	4,804,375	3,264,041	67.9	3,152,694	72,017	43.8	16,207	99
BC4732	IBE-BC4689	6,517,407	1,883,928	28.9	1,803,878	65,797	27.4	16,204	99
BC4850	IBE-BC4850	3,386,242	1,468,289	43.4	1,413,427	53,101	26.6	15,096	93
BC4810	IBE-BC4810	3,217,372	1,617,445	50.3	1,561,863	58,986	26.5	15,116	93
Average		2,798,706	1,296,782	36.5	1,239,291	39,536	23.8	9,958	61

Table S7. Sex corroboration results through detection of Y-chromosome sequences within genomic reads. Low-coverage samples (sequenced with less than 100,000 retained reads) could not be properly sex-assigned with this approach and are not represented.

Sample Code	Specimen Code	Retained reads	Hits chr Y	Y hits per million reads	Sex ddRAD
BC3755	IBE-BC2796	4,406,140	3,676	834	Male
BC4658		160,192	189	1,180	Male
BC2910	IBE-BC2796	1,880,090	1,635	870	Male
BC3724		323,697	215	664	Male
BC4571		543,293	565	1,040	Male
BC4554	IBE-BC2796	2,775,327	2,637	950	Male
BC4547	IBE-BC4689	1,336,689	1,046	783	Male
BC3748	IBE-BC4729	1,722,327	1,365	793	Male
BC3771		286,741	2	7	Female
BC3759		353,400	354	1,002	Male
BC3751	IBE-BC4810	2,724,128	1,787	656	Male
BC4693	IBE-BC4693	2,473,441	2,536	1,025	Male
BC4674	IBE-BC2796	1,506,203	1,670	1,109	Male
BC3058	IBE-BC2796	1,722,164	1,612	936	Male
BC4694		476,076	460	966	Male
BC4677	IBE-BC2796	1,320,049	1,499	1,136	Male
BC2796	IBE-BC2796	2,477,389	2,820	1,138	Male
BC4729	IBE-BC4729	1,212,466	1,184	977	Male
BC3048		133,189	125	939	Male
BC4689	IBE-BC4689	1,497,808	2,008	1,341	Male
BC4745	IBE-BC4729	4,479,618	5,168	1,154	Male
BC4855	IBE-BC4729	1,701,972	2,176	1,279	Male
BC4808	IBE-BC4689	3,264,041	3,283	1,006	Male
BC4732	IBE-BC4689	1,883,928	1,746	927	Male
BC4850	IBE-BC4850	1,468,289	3	2	Female
BC4810	IBE-BC4810	1,617,445	1,594	986	Male

Table S8. Genotyping error rates found in hair samples by comparing them with tissue samples.

Specimen code	Sample code	Allelic dropout rate	False allele rate	Sum error rate
IBE-BC2796	BC3755	0.09	0.03	0.12
	BC2910	0.11	0.05	0.16
	BC4554	0.12	0.04	0.16
	BC4674	0.11	0.07	0.18
	BC3058	0.12	0.06	0.19
	BC4677	0.07	0.06	0.13
	BC2796	0.07	0.05	0.12
IBE-BC4689	BC4547	0.08	0.09	0.16
	BC4689	0.08	0.07	0.15
	BC4808	0.04	0.04	0.09
	BC4732	0.04	0.06	0.10
IBE-BC4729	BC3748	0.05	0.07	0.12
	BC4729	0.05	0.05	0.10
	BC4745	0.02	0.02	0.04
	BC4855	0.04	0.02	0.06
Average		0.07	0.05	0.13

Table S9. COLONY results with mixed samples showing the probability of groups of samples inferred to belong to the same individual. Codes shown in bold indicate samples used in different proportions to create bioinformatically mixed samples. The mixed individuals are shown with the codes of the two source individuals and the percentage of reads of each of them. For example, sample "BC4693(25%)-BC4745(75%)" was generated with 25% reads of sample BC4693 and 75% reads of sample BC4745. Note that groups 2, 5 and 9 are inferred with 0 probability and should not be considered as a new individual.

Group	Probability	Members
1	1.000	BC3748, BC4729, **BC4745**, BC4855, BC4693(10%)-BC4745(90%), BC4693(25%)-BC4745(75%)
2	0.000	BC4693(50%)-BC4745(50%)
3	1.000	**BC4693**, BC4693(75%)-BC4745(25%), BC4693(90%)-BC4745(10%)
4	1.000	BC4547, BC4689, **BC4808**, BC4732, BC2796(10%)-BC4808(90%), BC2796(25%)-BC4808(75%)
5	0.000	BC2796(50%)-BC4808(50%)
6	1.000	BC3755, BC2910, BC4554, BC4674, BC3058, BC4677, **BC2796**, BC2796(75%)-BC4808(25%), BC2796(90%)-BC4808(10%)
7	1.000	**BC4810**, BC4850(10%)-BC4810(90%), BC4850(25%)-BC4810(75%)
8	0.754	**BC4850**, BC4850(50%)-BC4810(50%), BC4850(75%)-BC4810(25%), BC4850(90%)-BC4810(10%)
9	0.000	BC3751

Table S10. Individual inbreeding coefficient of hair and tissue replicates grouped by specimen.

Specimen code	Sample code	Inbreeding coefficient
IBE-BC2796	BC3755 (Hair)	0.04
	BC2910 (Hair)	0.14
	BC4554 (Hair)	0.15
	BC4674 (Hair)	0.08
	BC3058 (Hair)	0.13
	BC4677 (Hair)	0.00
	BC2796 (Hair)	0.00
	BC3939 (Tissue)	0.00
IBE-BC4689	BC4547 (Hair)	0.02
	BC4689 (Hair)	0.00
	BC4808 (Hair)	0.00
	BC4732 (Hair)	0.00
	BC4012 (Tissue)	0.00
IBE-BC4729	BC3748 (Hair)	0.19
	BC4729 (Hair)	0.14
	BC4745 (Hair)	0.12
	BC4855 (Hair)	0.18
	BC3179 (Tissue)	0.09
